# Associations of a perceived parental-rejection experience with self-harm or death thoughts in adolescents: evidence from symptom network analysis and structural equation modeling

**DOI:** 10.3389/fpsyt.2026.1927040

**Published:** 2026-08-28

**Authors:** Jiajun Yin, Mengmeng Fan, Xia Sun, Yingying Ji, Xiaoyan He

**Affiliations:** 1 Department of Psychiatry, The Affiliated Mental Health Center of Jiangnan University, Wuxi, China; 2 School of Logistics Management, Wuxi Institute of Communications Technology, Wuxi, China

**Keywords:** childhood trauma, death thoughts, depressive symptoms, generalized anxiety symptoms, parental rejection, self-harm thoughts, structural equation modeling, symptom network

## Abstract

**Background:**

Childhood trauma (CT) is associated with depression, anxiety, and suicide-related thoughts in adolescents. However, the item-level associations linking trauma-related experiences with internalizing symptoms and thoughts of self-harm or death remain unclear.

**Methods:**

We conducted a cross-sectional analysis of 2,413 adolescents who completed the Childhood Trauma Questionnaire-Short Form (CTQ-SF), Patient Health Questionnaire-9 (PHQ-9), and Generalized Anxiety Disorder-7 (GAD-7). Symptom-level associations were examined using a Spearman-EBICglasso network, with a polychoric network as a sensitivity analysis. CTQ-SF item 8 was interpreted descriptively as a perceived parental-rejection experience. PHQ-9 items 1–8 indicated latent depressive symptoms, GAD-7 items 1–7 indicated latent generalized anxiety symptoms, and PHQ-9 item 9 represented thoughts of being better off dead or self-harm. Four cross-sectional structural equation model (SEM) specifications were compared using maximum likelihood (ML), with MLR and WLSMV sensitivity analyses.

**Results:**

Childhood trauma, depressive symptoms, and generalized anxiety symptoms formed distinct but interconnected network communities. CTQ-SF item 8 ranked seventh in bridge strength in the primary network and remained among the leading bridge items in the polychoric network. The correlated-factors SEM showed the best primary fit. Under ML, item 8 was associated with depressive symptoms (standardized β = 0.439, p < 0.001), generalized anxiety symptoms (standardized β = 0.377, p < 0.001), and item 9 directly (standardized β = 0.077, p = 0.012). The depressive-symptom association with item 9 was larger (standardized β = 0.562, p < 0.001) than the generalized-anxiety association (standardized β = 0.111, p = 0.073). MLR reproduced this pattern, whereas WLSMV identified a smaller but statistically significant generalized-anxiety association (standardized β = 0.166, p = 0.001).

**Conclusions:**

A perceived parental-rejection experience emerged as a salient bridge item linking childhood trauma with internalizing symptoms and thoughts of self-harm or death. Depressive symptoms showed the larger and more consistent conditional association with item 9, whereas the generalized-anxiety association was smaller and varied by estimator. These findings support further investigation of family relational experiences in adolescent mental-health assessment and prevention research.

## Introduction

Childhood trauma (CT) is widely recognized as a major contributor to the development of internalizing psychopathology, including depression, anxiety, and suicidality, with enduring negative consequences for affected individuals (1, 2). CT encompasses exposure to life-threatening or adverse events during childhood, such as physical, emotional, and sexual abuse, as well as physical and emotional neglect (3). It has become a global concern: World Health Organization (WHO) surveys across 21 countries estimate that more than one-third of the population has experienced CT (4). In university settings, more recent data indicate that approximately 76% of students have endured at least one adverse childhood experience (5).

Epidemiological evidence consistently demonstrates significant associations between CT and psychiatric symptoms (6, 7), with psychological consequences often persisting into adulthood or even lifelong (8, 9). Numerous investigations indicate that CT is associated with depressive symptoms in both adolescence (10) and adulthood (11). Meta-analyses further suggest that the strength of these associations varies according to trauma type and depression severity (6, 7). Among adolescents in vocational or college settings, academic demands, pressure to succeed, and uncertainty about future career plans may co-occur with prior adversity and heighten vulnerability to mental-health problems (12–15).

Furthermore, CT and trauma-related interpersonal experiences are associated with suicidal ideation and self-harm-related thoughts (16–18). A comprehensive meta-analysis demonstrated that childhood sexual, physical, and emotional abuse were associated with substantially greater odds of suicidal ideation and attempts (17). Prior studies have also reported that depressive symptoms and emotion-related processes statistically account for part of the association between childhood trauma and suicidal ideation (18). Adolescence, as a sensitive developmental stage, may therefore represent a critical period in which adverse childhood experiences are linked to internalizing symptoms and suicide-related thoughts.

However, several gaps remain. First, symptom network studies can identify central and bridge items, but relatively few studies have examined how these item-level findings correspond to broader latent-variable models. Second, depressive and generalized anxiety symptoms are strongly interrelated, making it important to examine both their distinct and shared associations with self-harm or death thoughts. Third, it remains unclear whether particular trauma-related interpersonal experiences may help clarify the links between childhood trauma and adolescent internalizing symptoms.

To address these issues, the present study adopted a two-stage analytic strategy in a large sample of Chinese adolescents. We first used item-level network analysis to examine associations among childhood trauma, depressive symptoms, generalized anxiety symptoms, and PHQ-9 item 9 and to identify bridge items across domains. We then compared four cross-sectional SEM specifications centered on the trauma-related item identified in the network. Because the focal item emerged from the first-stage analysis, the SEM stage was treated as exploratory. This approach was intended to integrate symptom-level specificity with latent-variable modeling and to generate more focused hypotheses for future adolescent mental-health research.

## Methods

### Participants and data collection

This cross-sectional study was carried out in May 2023 at one vocational college in Wuxi, Jiangsu Province, China. Cluster sampling was implemented by recruiting students from selected intact teaching classes. Of 2,669 questionnaires distributed, 2,413 complete and valid questionnaires were retained (90.4%); 256 were excluded during pre-analysis screening because they were incomplete and/or did not meet the original survey validity criteria. Detailed mutually exclusive exclusion categories were not preserved in the archived dataset. No missing values remained for the core CTQ-SF, PHQ-9, or GAD-7 items in the final sample (**Supplementary Table 1**), and class identifiers were not retained in the analytic file. Data were collected anonymously and voluntarily through Wenjuanxing (www.sojump.com) (19, 20). Participants were informed of the study purpose, confidentiality, and right to withdraw. Completion and submission of the questionnaire were considered as online informed consent for participants aged 18 years or older and online informed assent for participants younger than 18 years. Because this was an anonymous, voluntary, minimal-risk online mental health screening survey conducted in an educational setting, the Ethics Committee approved the use of online informed assent for participants younger than 18 years without requiring separate written parental/legal guardian consent. The study protocol was approved by the Ethics Committee of the Wuxi Mental Health Center (approval no. WXMHCIRB2023LLky127).

The same survey cohort contributed to a previous report focused on sleep, depressive, and generalized anxiety symptom networks and sex-specific comparisons (21). The present study addresses a distinct trauma-focused question by incorporating CTQ-SF items and SEM.

### Measurements

#### The childhood trauma questionnaire-short form

CT was assessed using the Chinese version of the Childhood Trauma Questionnaire-Short Form (CTQ-SF), a self-report tool that evaluates childhood abuse and neglect experiences across five validated domains: emotional abuse, physical abuse, sexual abuse, emotional neglect, and physical neglect (22–24). The CTQ-SF consists of 28 items, each rated on a 5-point Likert scale, with scores ranging from 1 to 5 (1 = never, 2 = rarely, 3 = sometimes, 4 = often, 5 = very often). Seven items (e.g., Items 2, 5, 7, 13, 19, 26, 28) are reverse-coded. Three additional items (Items 10, 16, 22) assess response validity and are excluded from scoring (**Supplementary Table 1**). CTQ-SF item 8 reads, “I thought my parents wished I had never been born” (Chinese wording: “我觉得父母希望从来没有生过我”). We labeled this item as perceived parental rejection for interpretive clarity, while recognizing that it is a single item within the CTQ-SF rather than a validated multi-item parental rejection scale.

Higher scores indicate greater perceived CT. The scale is divided into five subscales, one for each type of trauma, and an overall score for total CT. Subscale scores range from 5 to 25, and the total trauma score (excluding validity items) ranges from 25 to 125. Individuals were categorized as having experienced moderate-to-severe trauma if they met established cutoffs in any subscale: emotional abuse ≥13, physical abuse ≥10, sexual abuse ≥8, emotional neglect ≥15, or physical neglect ≥10 (22, 23). This criterion was used to dichotomize trauma exposure in the present study. In the present study, the Cronbach’s α for this scale was 0.82, demonstrating good internal consistency.

#### Patient health questionnaire-9

Depressive symptoms were evaluated with the Chinese version of the Patient Health Questionnaire-9 (PHQ-9), a widely used self-report tool assessing symptoms during the preceding two weeks (25, 26). The scale covers nine symptoms: anhedonia, depressed mood, sleep problems, fatigue, appetite changes, guilt, concentration difficulties, psychomotor changes, and thoughts that one would be better off dead or of self-harm. Each item is scored from 0 (not at all) to 3 (nearly every day), with higher scores indicating greater depressive symptom severity (**Supplementary Table 1**). In the SEM, PHQ-9 items 1–8 indicated the latent depressive symptom factor, whereas item 9 was analyzed separately as an observed indicator of self-harm or death thoughts rather than as a comprehensive suicidality measure (27). The PHQ-9 demonstrated excellent internal consistency in this sample (Cronbach’s α = 0.93).

#### Generalized anxiety disorder scale-7

Generalized anxiety symptoms were measured using the validated Chinese version of the Generalized Anxiety Disorder Scale-7 (GAD-7), which assesses nervousness, uncontrollable worry, excessive worry, difficulty relaxing, restlessness, irritability, and fear (28, 29). Items are scored from 0 (not at all) to 3 (almost every day), with higher total scores reflecting greater generalized anxiety symptom severity (**Supplementary Table 1**). The GAD-7 was selected because it is brief, validated in Chinese adolescents, and suitable for large-scale surveys. It showed excellent internal consistency in this sample (Cronbach’s α = 0.94).

### Statistical analysis

#### Network estimation

The primary network was estimated in qgraph using a Spearman correlation matrix followed by EBICglasso regularization with a tuning parameter of γ = 0.5 (30, 31). Spearman correlations were selected as a rank-based approach for the skewed ordinal item responses. As an ordinal-data sensitivity analysis, EBICglasso was re-estimated from a polychoric correlation matrix using the same sample size and tuning parameter. Agreement between the two networks was examined using edge overlap, edge-weight correlations, bridge-rank correlations, the CTQ8-PHQ9 edge, and the rank of CTQ8 two-step bridge expected influence.

To evaluate the structural importance of nodes, we employed *qgraph* (v1.9.8) together with *networktools* (v1.6.0) (32, 33). Centrality metrics were calculated to capture both the strength and connectivity of each node, thereby illuminating the overall network organization. Node importance was first quantified using strength centrality, defined as the sum of the absolute weights of its connections. Because this approach may obscure the role of nodes involved in negative associations, expected influence was also computed as a complementary index, accounting for both positive and negative connections.

Furthermore, bridge centrality was used to determine the extent to which individual nodes served as links across different symptom clusters. Two-step bridge-expected influence provided an additional indicator of how strongly each node connected with nodes from other clusters, highlighting cross-domain statistical links between trauma-related items and internalizing symptoms.

#### Network stability and accuracy

We evaluated the stability and accuracy of the estimated network using the *bootnet* (v1.6) package in R. Non-parametric bootstrapping was applied to obtain edge weight estimates and corresponding 95% confidence intervals. In addition, the stability of centrality indices was examined through a case-dropping bootstrap approach, which progressively omits subsets of participants to assess the robustness of centrality estimates. A correlation stability coefficient (CS-coefficient) above 0.25 was considered acceptable, whereas values greater than 0.50 indicated strong stability. To further evaluate network reliability, bootstrapped difference tests with 1,000 iterations were performed to compare expected influence and bridge-expected influence across network properties.

### Structural equation modeling

SEM was used to compare cross-sectional representations of the associations among CTQ-SF item 8, latent depressive symptoms, latent generalized anxiety symptoms, and PHQ-9 item 9. CTQ-SF item 8, identified as a prominent bridge item in the preceding network analysis, served as the focal observed predictor. PHQ-9 items 1–8 indicated the depressive symptom factor, GAD-7 items 1–7 indicated the generalized anxiety symptom factor, and PHQ-9 item 9 was the observed outcome. Sex and age were included as covariates for all endogenous variables. Because the focal item emerged from the network stage, the SEM analyses were considered exploratory.

Four theoretically plausible parameterizations were compared. Models 1 and 2 represented alternative sequential statistical orderings of the depressive and generalized anxiety symptom factors. Model 3 specified uncorrelated parallel indirect associations. Model 4 specified correlated symptom factors: CTQ-SF item 8 was associated with both factors, the factors were allowed to covary, and both were associated with PHQ-9 item 9.

The primary SEM used maximum likelihood (ML) estimation with 5,000 bootstrap draws to derive standard errors and 95% confidence intervals. Model fit was evaluated using CFI, TLI, RMSEA, and SRMR; AIC, BIC, and SABIC were used to compare the four focal-item parameterizations. Model 4 was additionally estimated using robust maximum likelihood (MLR) and weighted least squares mean- and variance-adjusted estimation (WLSMV). All PHQ-9 and GAD-7 items were specified as ordered in the WLSMV analysis. The SEM and sensitivity analyses were conducted in R 4.6.1 using lavaan 0.7-2.

Discriminant validity was examined using composite reliability (CR), average variance extracted (AVE), and the heterotrait-monotrait ratio (HTMT). CR and AVE were calculated from standardized Model 4 loadings; HTMT was computed from raw interitem correlations, with a Spearman-based value as a sensitivity check. Two-factor and one-factor internalizing measurement models were also compared using WLSMV.

Given the cross-sectional design, the SEM analyses were intended to compare alternative statistical representations of the observed associations.

## Results

### Characteristics of the participants and scales

The final sample comprised 2,413 adolescents aged 14–19 years (mean = 17.15, SD = 1.14), including 1,524 males (63.2%) and 889 females (36.8%). No missing values were present in the 41 network nodes or core SEM variables.

For PHQ-9 item 9, 2,192 participants (90.8%) reported no self-harm/death thoughts, 167 (6.9%) reported such thoughts on several days, 40 (1.7%) reported them on more than half the days, and 14 (0.6%) reported them nearly every day. In the network and SEM analyses, PHQ-9 item 9 was treated as an observed item-level indicator rather than as a clinical diagnosis or comprehensive suicidality measure.

**Table 1** and **Supplementary Table 1** summarize the scale and item-level characteristics. The mean CTQ-SF total score was 40.64 (SD = 10.58), with a small male-female difference (41.08 vs. 39.89, p < 0.05). Females reported higher PHQ-9 (2.54 vs. 1.69, p < 0.001) and GAD-7 scores (2.52 vs. 1.62, p < 0.001).

**Table 1:** Characteristics of the CTQ-SF, PHQ-9 and GAD-7 item-level symptoms of the participants.

Reference name	All adolescents (n = 2413)	Male (n = 1524)	Female (n = 889)	*z/t*
CTQ-SF				
CTQ-SF total scores (Mean ± SD)	40.64 ± 10.58	41.08 ± 10.98	39.89 ± 9.80	-2.01*
Emotional abuse (1/2) ^a^ (n)	2316/97	1465/59	851/38	0.24
Physical abuse (1/2) ^a^ (n)	2259/154	1410/114	849/40	8.35**
Sexual abuse (1/2) ^a^ (n)	2274/139	1413/111	861/28	17.68***
Emotional neglect (1/2) ^a^ (n)	1481/932	918/606	563/326	2.27
Physical neglect (1/2) ^a^ (n)	1395/1018	845/679	550/339	9.49**
CTQ-SF (1/2) ^a^ (n)	1085/1328	654/870	431/458	7.03**
PHQ-9				
PHQ-9 total scores (Mean ± SD)	2.00 ± 3.84	1.69 ± 3.55	2.54 ± 4.24	-6.62***
PHQ-9 (1/2/3/4/5) ^a^ (n)	2000/292/73/30/18	1303/156/43/13/9	697/136/30/17/9	22.27*
GAD-7				
GAD-7 total scores (Mean ± SD)	1.95 ± 3.47	1.62 ± 3.27	2.52 ± 3.73	-8.47***
GAD-7 (1/2/3/4) ^a^ (n)	1977/347/53/36	1295/182/26/21	682/165/27/15	26.66***

*p < 0.05, **p < 0.01, ***p < 0.001.

a, The Chi-square test was used to assess gender differences, while for continuous variables the Mann–Whitney U test was applied.

The CTQ-SF (1/2) was used to categorize overall childhood trauma: 1 = no significant childhood trauma, 2 = moderate-to-severe trauma. Similarly, each CTQ-SF subscale was dichotomized: Emotional abuse (1 = no emotional abuse, 2 = presence of emotional abuse), Physical abuse (1 = no physical abuse, 2 = presence of physical abuse), Sexual abuse (1 = no sexual abuse, 2 = presence of sexual abuse), Emotional neglect (1 = no emotional neglect, 2 = presence of emotional neglect), and Physical neglect (1 = no physical neglect, 2 = presence of physical neglect).

The PHQ-9 (1/2/3/4/5) categorizes depressive symptom severity: 1 = no depression, 2 = mild, 3 = moderate, 4 = moderately severe, 5 = severe.

The GAD-7 (1/2/3/4) categorizes anxiety severity: 1 = no anxiety, 2 = mild, 3 = moderate, 4 = severe.

The notation “n” refers to the number of participants.

### Estimation of the CT-depression–anxiety symptom network

**Figure 1:**
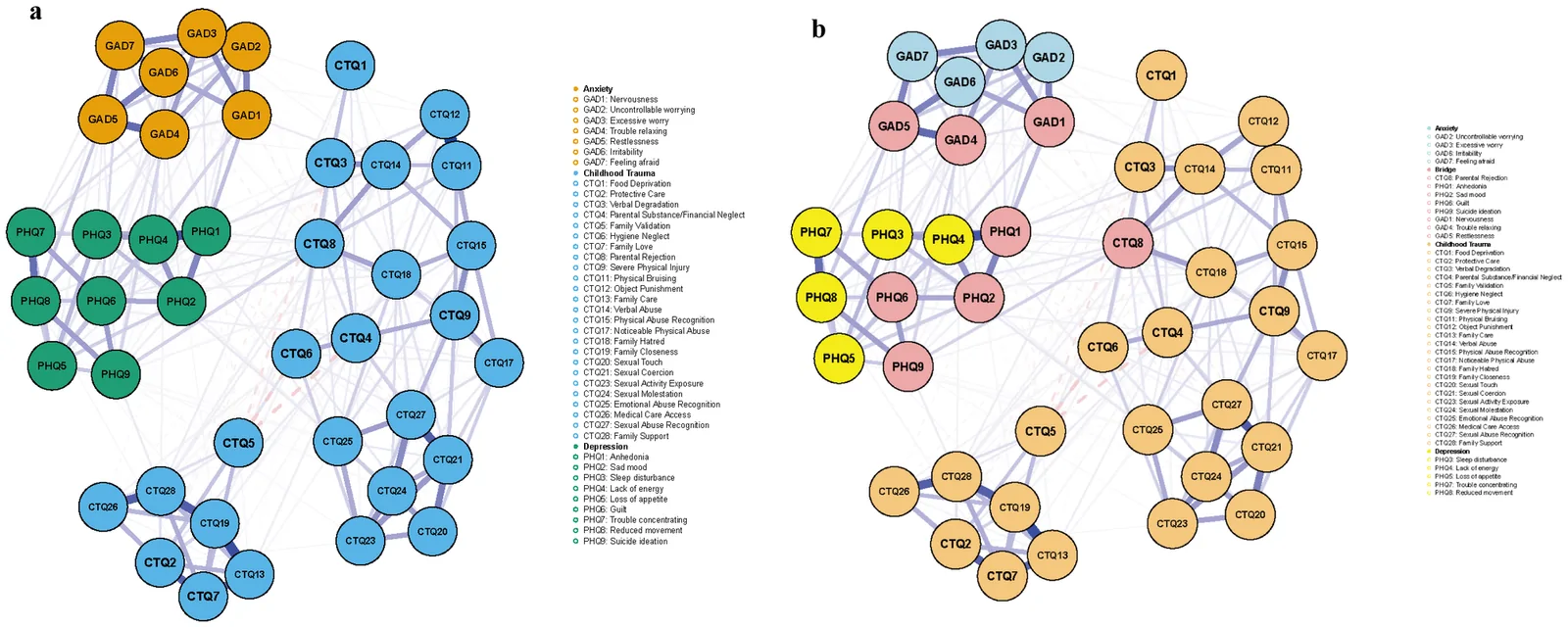
Network structure and bridge network of childhood trauma, depressive, and generalized anxiety symptoms. **(a)** Estimated network structure showing associations among childhood trauma, depression, and anxiety symptoms. **(b)** Bridge centrality analysis identifying key symptoms linking the three domains. Nodes are grouped and color-coded by symptom clusters. Blue edges represent positive associations, while red edges denote negative associations. The thickness of each edge reflects the strength of the relationship, with thicker edges corresponding to stronger associations.

The primary Spearman-EBICglasso network contained 41 nodes and 314 non-zero edges of 820 possible edges (density = 38.3%; mean edge weight = 0.022). The CTQ-SF, PHQ-9, and GAD-7 communities were visually distinguishable but interconnected (**Figure 1**; **Supplementary Table 2**). The strongest cross-community edges included PHQ2-GAD1, PHQ8-GAD5, and CTQ8-PHQ9. We refer to CTQ8 descriptively as the parental-rejection-related item and to PHQ9 as thoughts of being better off dead or self-harm.

**Figure 2:**
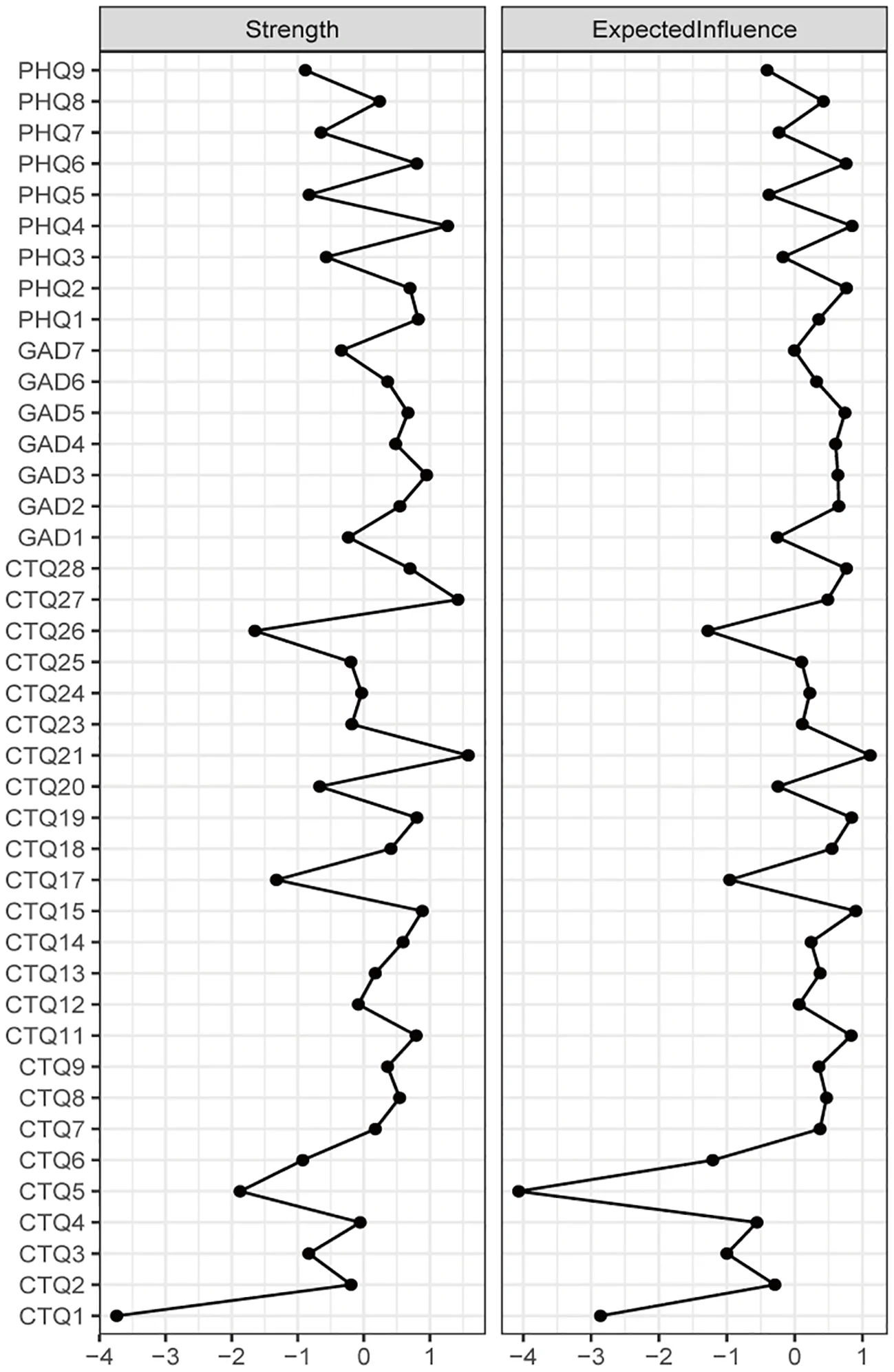
Node centrality indices of the childhood trauma-depression-anxiety symptom network. Node strength and expected influence across all participants. Higher standardized values indicate greater within-network importance under the primary Spearman-EBICglasso specification.

**Figure 3:**
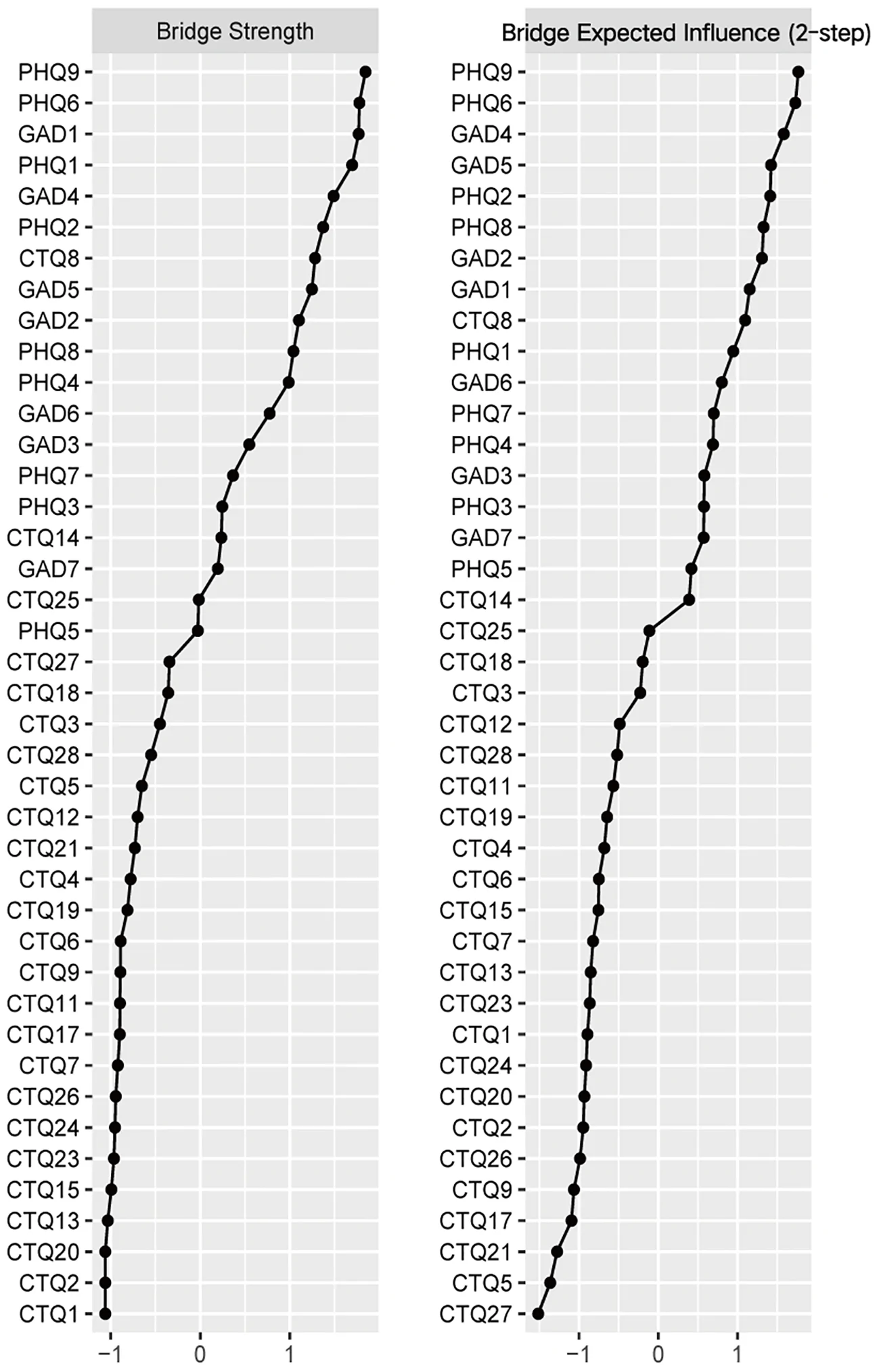
Bridge centrality indices of the childhood trauma-depression-anxiety symptom network. Bridge strength and two-step bridge expected influence across all participants. Higher standardized values indicate stronger cross-community connectivity under the primary Spearman-EBICglasso specification.

Node centrality analysis (**Figure 2**) identified CTQ21, PHQ4, and GAD3 as the highest-strength items within their respective communities. Bridge centrality analysis (**Figure 3**) identified PHQ9, PHQ6, GAD1, PHQ1, GAD4, PHQ2, CTQ8, and GAD5 as the eight highest-ranking nodes in bridge strength. CTQ8 ranked seventh of 41 nodes in bridge strength and ninth in two-step bridge expected influence.

### Network accuracy analysis results

In the primary network, nonparametric bootstrap intervals were relatively narrow for most edge weights, and the correlation-stability coefficients for expected influence and bridge expected influence were both 0.75 (**Supplementary Figure 1**), indicating good stability of the centrality estimates.

In the polychoric sensitivity network, the CTQ8-PHQ9 edge persisted (0.075 in the primary network and 0.113 in the polychoric network), and CTQ8 remained among the leading two-step bridge-expected-influence items (rank 9 and rank 8, respectively). The networks shared 262 edges (Jaccard = 0.437); edge-weight correlations were 0.754 (Pearson) and 0.628 (Spearman), and two-step bridge expected-influence and bridge-strength rank correlations were 0.927 and 0.706. Full results are provided in **Supplementary Table 3**.

### Comparison of competing structural equation models

Among the four CTQ-SF item 8 parameterizations, Model 4, the correlated-factors model, had the best primary fit (CFI = 0.949, TLI = 0.939, RMSEA = 0.068, SRMR = 0.027) and the lowest AIC, BIC, and SABIC (**Table 2**). Models 1 and 2 produced identical fit statistics, indicating that their alternative statistical orderings could not be distinguished in these cross-sectional data.

**Table 2:** Fit indices for competing SEMs.

Model	Description	*χ²*	*df*	CFI	TLI	RMSEA	SRMR	AIC	BIC	SABIC
Model 1	Alternative sequential decomposition: CTQ8 -> depressive symptoms -> generalized anxiety symptoms -> PHQ9	1747.891	142	0.948	0.938	0.068	0.027	34503.33	34839.07	34654.79
Model 2	Alternative sequential decomposition: CTQ8 -> generalized anxiety symptoms -> depressive symptoms -> PHQ9	1747.891	142	0.948	0.938	0.068	0.027	34503.33	34839.07	34654.79
Model 3	Uncorrelated parallel indirect-association model	3837.624	142	0.880	0.858	0.104	0.233	36593.07	36928.81	36744.53
**Model 4**	Correlated-factors indirect-association model	**1727.452**	**141**	**0.949**	**0.939**	**0.068**	**0.027**	**34484.89**	**34826.42**	**34638.97**

CTQ8, parental-rejection-related item from the CTQ-SF; PHQ9, PHQ-9 item assessing thoughts of being better off dead or self-harm. Models 1 and 2 are statistically indistinguishable sequential decompositions; Model 3 is an uncorrelated parallel indirect-association model; Model 4 is a correlated-factors model. Lower AIC/BIC/SABIC values indicate better relative fit only among these four structurally comparable focal-item parameterizations. Bold values indicate the best-fitting model among the competing models.

### Final SEM linking CTQ-SF item 8, depressive symptoms, generalized anxiety symptoms, and PHQ-9 item 9

**Figure 4:**
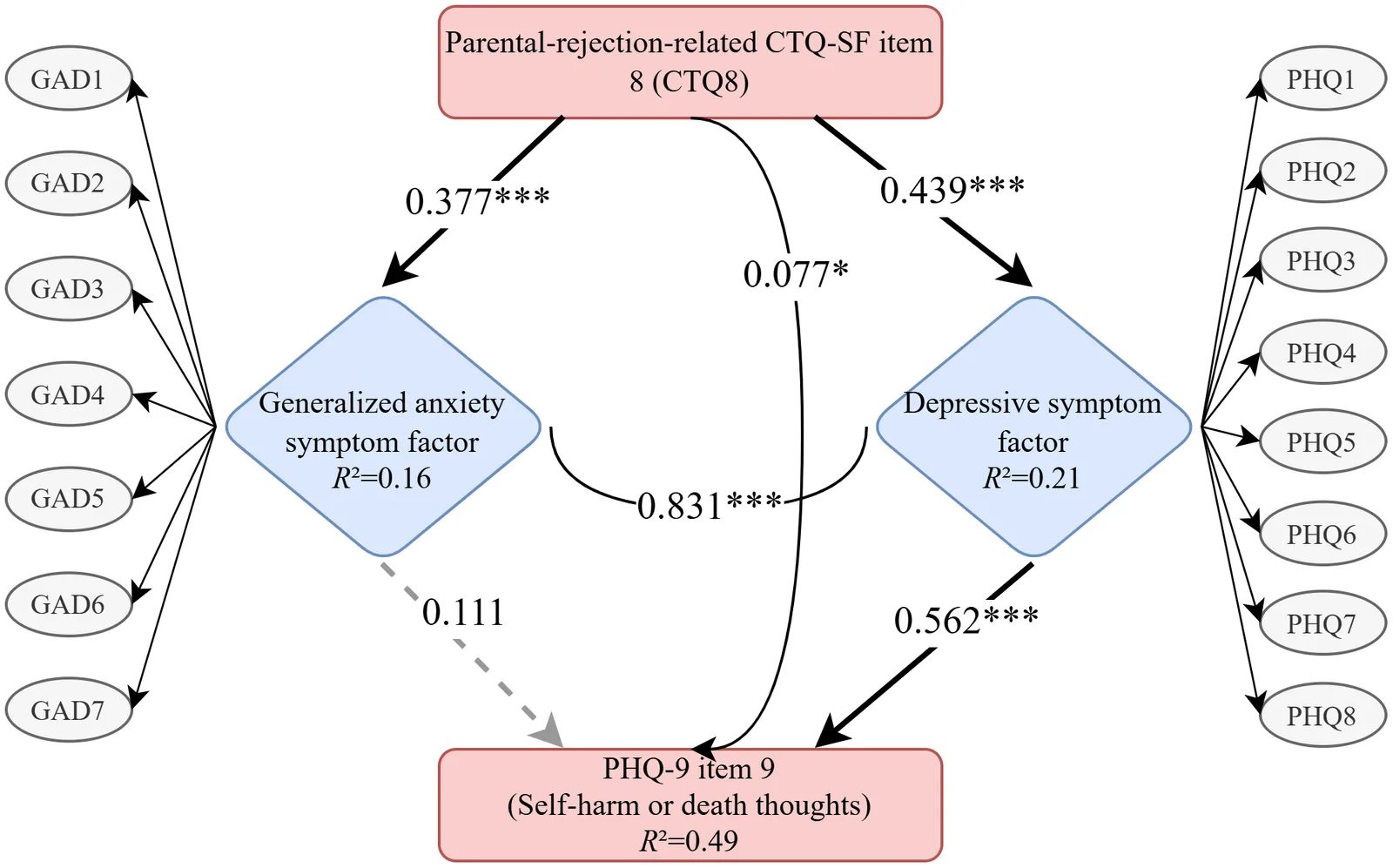
Final cross-sectional SEM linking the parental-rejection-related CTQ-SF item, depressive symptoms, generalized anxiety symptoms, and PHQ-9 item 9. The image displays standardized coefficients from the primary ML model with bootstrap inference. Solid arrows indicate statistically significant coefficients in the primary model; the dashed generalized-anxiety-to-PHQ9 arrow was not significant under ML/MLR but was significant under WLSMV. The symptom factors were allowed to covary. Sex and age covariates are omitted for visual clarity. Explained variance was 21% for depressive symptoms, 16% for generalized anxiety symptoms, and 49% for PHQ-9 item 9. ***p < 0.001, *p < 0.05. The curved line without arrowheads represents the standardized residual correlation between the latent depressive symptom factor and the latent generalized anxiety symptom factor.

Model 4 was retained as the best-fitting cross-sectional parameterization of the focal-item model (**Figure 4**; **Table 3**). In the primary ML analysis, CTQ-SF item 8 was associated with latent depressive symptoms (standardized *β* = 0.439, *p* < 0.001) and latent generalized anxiety symptoms (standardized *β* = 0.377, *p* < 0.001). The factors showed a strong residual association (*r* = 0.831, *p* < 0.001), indicating substantial shared internalizing variance.

**Table 3:** Standardized estimates for the primary SEM (model 4: correlated factors).

Path/statistical association	B	SE	z	p	95% bootstrap CI	Std. β
Depressive symptoms ← CTQ-SF item 8	0.264	0.020	13.123	<0.001	0.226 to 0.304	0.439
Generalized anxiety symptoms ← CTQ-SF item 8	0.238	0.021	11.371	<0.001	0.196 to 0.280	0.377
PHQ-9 item 9 ← Depressive symptoms	0.499	0.064	7.808	<0.001	0.374 to 0.628	0.562
PHQ-9 item 9 ← Generalized anxiety symptoms	0.094	0.053	1.792	0.073	-0.010 to 0.195	0.111
PHQ-9 item 9 ← CTQ-SF item 8 (direct effect)	0.041	0.016	2.518	0.012	0.010 to 0.073	0.077
Depressive symptoms ~~ Generalized anxiety symptoms	0.157	0.012	13.517	<0.001	0.134 to 0.180	0.831
Indirect statistical association: CTQ-SF item 8 → Depressive symptoms → PHQ-9 item 9	0.132	0.020	6.539	<0.001	0.094 to 0.173	0.247
Indirect statistical association: CTQ-SF item 8 → Generalized anxiety symptoms → PHQ-9 item 9	0.022	0.013	1.775	0.076	-0.003 to 0.047	0.042
Total indirect statistical association	0.154	0.016	9.738	<0.001	0.124 to 0.187	0.289
Total statistical association of CTQ-SF item 8 on PHQ-9 item 9	0.195	0.020	9.709	<0.001	0.157 to 0.235	0.366

Model R²: Depression = 0.21; Anxiety = 0.16; PHQ9 = 0.49.

CTQ8, the parental-rejection-related CTQ-SF item; PHQ9, PHQ-9 item 9 (thoughts of being better off dead or self-harm). B, SE, z, p, and 95% confidence intervals are from the primary ML model with bootstrap resampling; Std. beta is Std.all. Sex and age were included as covariates but are omitted for brevity. Indirect coefficients are reported as cross-sectional statistical associations.

In the primary ML model, latent depressive symptoms were associated with PHQ-9 item 9 (standardized β = 0.562, p < 0.001), whereas the smaller generalized-anxiety coefficient did not reach the prespecified significance threshold (standardized β = 0.111, p = 0.073). CTQ-SF item 8 retained a small direct association with item 9 (standardized β = 0.077, p = 0.012). The indirect statistical association through depressive symptoms was significant (standardized β = 0.247, p < 0.001), whereas the anxiety-specific indirect association was not (standardized β = 0.042, p = 0.076). The total indirect association (standardized β = 0.289, p < 0.001) and total association (standardized β = 0.366, p < 0.001) were also significant.

Composite reliability and average variance extracted were 0.922 and 0.598 for depressive symptoms and 0.940 and 0.691 for generalized anxiety symptoms. The HTMT ratio was 0.860 using Pearson correlations and 0.823 using Spearman correlations. In WLSMV CFA, the two-factor model fit better than the one-factor internalizing model (CFI = 0.954 vs. 0.881; TLI = 0.946 vs. 0.862; RMSEA = 0.104 vs. 0.167; SRMR = 0.022 vs. 0.046), although the latent factor correlation was high (r = 0.900) (**Supplementary Table 5**).

Estimator sensitivity was also examined for Model 4. MLR reproduced the primary standardized coefficients and yielded robust fit indices of CFI = 0.956, TLI = 0.948, RMSEA = 0.063, and SRMR = 0.027. WLSMV also fit well (CFI = 0.987, TLI = 0.989, RMSEA = 0.046, SRMR = 0.027) and retained all focal coefficient directions. Under WLSMV, the generalized-anxiety association with PHQ-9 item 9 was smaller than the depressive association but statistically significant (standardized β = 0.166, p = 0.001, versus standardized β = 0.667, p < 0.001). The anxiety-specific indirect association was also significant under WLSMV (standardized β = 0.056, p = 0.002) (**Supplementary Table 4**).

## Discussion

This study combined symptom network analysis and SEM to examine item-level and construct-level associations among childhood trauma, internalizing symptoms, and thoughts of self-harm or death in adolescents. Two findings are particularly noteworthy. First, CTQ-SF item 8, describing a perceived parental-rejection experience, emerged as a prominent bridge item in the primary network and remained among the leading bridge items in the polychoric sensitivity network. Second, the correlated-factors SEM provided the best primary fit, with depressive symptoms showing a larger and more consistent association with PHQ-9 item 9 than generalized anxiety symptoms.

The network findings are broadly consistent with trauma-informed network studies showing cross-domain links among maltreatment, depressive, anxiety, and self-harm-related items (34–36). The polychoric sensitivity analysis reinforced the focal finding: the CTQ8-PHQ9 edge persisted and CTQ8 remained among the leading two-step bridge-expected-influence items. At the broader network level, the moderate edge overlap indicates that correlation estimation can influence the detailed topology of regularized symptom networks.

The SEM sensitivity analyses further refined the interpretation. MLR reproduced the primary ML pattern, whereas WLSMV identified a statistically significant generalized-anxiety association with PHQ-9 item 9. Across estimators, however, the depressive association remained larger. Together with the strong residual covariance and high latent correlation between the symptom factors, this pattern suggests that depression and anxiety share substantial internalizing variance while retaining differences in their conditional associations with self-harm or death thoughts.

From a theoretical perspective, the prominence of CTQ-SF item 8 is consistent with Interpersonal Acceptance-Rejection Theory, which links perceived parental nonacceptance with emotional maladjustment and internalizing symptoms (37). The item may capture a particularly salient interpersonal experience within the broader emotional-abuse domain. Because it is a single CTQ-SF item rather than a validated multi-item parental-rejection measure, this interpretation provides a focused hypothesis for further study.

The present results also align with prior work on trauma and suicidality. Angelakis et al. showed that multiple forms of childhood maltreatment are associated with substantially elevated odds of suicidal ideation and attempts in young people (17). Gong et al., in a large Chinese adolescent sample, likewise reported that childhood maltreatment was associated with suicidal behavior and that depressive symptoms shaped these associations (38). Laghaei et al. also found that depressive symptoms and emotion-regulation difficulties statistically accounted for part of the association between childhood trauma and suicidal ideation (18). Our findings extend this literature by showing how item-level and latent-variable analyses can jointly identify specific interpersonal experiences for further investigation.

Clinically, these item-level findings may help focus future family-context assessment and school mental-health research. Asking about perceived parental nonacceptance alongside broader trauma exposure, depressive and anxiety symptoms, and self-harm or death thoughts may help identify patterns that warrant fuller assessment. Such inquiry should complement validated multi-item trauma and suicidality measures rather than function as a stand-alone screening approach.

Several limitations should be acknowledged. First, the cross-sectional, self-report design precludes temporal or causal inference and may be affected by recall and common-method bias; in addition, CTQ-SF item 8 was selected and modeled in the same sample, so independent replication is needed. Second, PHQ-9 item 9 combines passive death wishes and self-harm thoughts and shares an instrument with the depressive indicators, while the high depression-anxiety overlap and estimator-sensitive anxiety coefficient complicate separation of their specific associations. Third, the single vocational-college sample, unequal sex distribution, unavailable class identifiers, and unmeasured family or clinical covariates may limit generalizability and precision. Finally, the anonymous survey did not permit individual follow-up after endorsement of item 9. Longitudinal, externally replicated studies using dedicated suicidality measures and prospective safety procedures are warranted.

## Conclusions

The present study suggests that a perceived parental-rejection experience may represent a salient trauma-related bridge item associated with internalizing symptoms and thoughts of self-harm or death in adolescents. Depressive symptoms showed the larger and more consistent conditional association, whereas the generalized-anxiety association was smaller and varied across estimators. Taken together, the symptom-level and construct-level findings may inform future research on family relational experiences and adolescent mental-health risk.

## Data Availability

The original contributions presented in the study are included in the article/**Supplementary Material**. Further inquiries can be directed to the corresponding author.
