# Supplementary material for: Associations of a perceived parental-rejection experience with self-harm or death thoughts in adolescents: evidence from symptom network analysis and structural equation modeling
Source: Front Psychiatry. 2026 Aug 28;17:1927040. doi: 10.3389/fpsyt.2026.1927040 (PMC13562260; doi:10.3389/fpsyt.2026.1927040)
Supplement: Supplementary file 1 [file Supplementaryfile1.docx]

**Supplementary Materials**

**Table S1** **Characteristics of the CTQ-SF, PHQ-9, and GAD-7 items**

| Items | Reference name | |
| --- | --- | --- |
|  |  |  |
| CTQ-SF |  |  |
| I didn't have enough to eat | CTQ1 | Food Deprivation |
| I had someone to take care of and protect me | CTQ2 | Protective Care |
| Family members called me "stupid," "lazy," or "ugly" | CTQ3 | Verbal Degradation |
| My parents were too intoxicated or preoccupied with substance use/spending to care for the family | CTQ4 | Parental Substance/Financial Neglect |
| Someone in my family made me feel special or important | CTQ5 | Family Validation |
| I had to wear dirty clothes | CTQ6 | Hygiene Neglect |
| I felt loved by family members | CTQ7 | Family Love |
| I thought my parents wished I had never been born | CTQ8 | Parental-rejection-related item |
| I was hurt so badly by someone in my family that I required medical attention | CTQ9 | Severe Physical Injury |
| I wouldn't have wanted to change anything about my family | CTQ10 | Family Satisfaction |
| Family members hit me hard enough to leave bruises or marks | CTQ11 | Physical Bruising |
| I was punished with a belt, cord, board, or other hard object | CTQ12 | Object Punishment |
| Family members looked out for each other | CTQ13 | Family Care |
| People in my family said hurtful or insulting things to me | CTQ14 | Verbal Abuse |
| I believe I was physically abused | CTQ15 | Physical Abuse Recognition |
| I had a happy childhood | CTQ16 | Childhood Quality |
| I was beaten badly enough to be noticed by teachers, neighbors, or medical professionals | CTQ17 | Noticeable Physical Abuse |
| I felt that someone in my family hated me | CTQ18 | Family Hatred |
| Family members felt close to each other | CTQ19 | Family Closeness |
| Someone tried to touch me sexually or made me touch them | CTQ20 | Sexual Touch |
| Someone threatened or coerced me to engage in sexual acts | CTQ21 | Sexual Coercion |
| My family was the best in the world | CTQ22 | Family Idealization |
| Someone tried to make me do or watch sexual activities | CTQ23 | Sexual Activity Exposure |
| I was sexually molested | CTQ24 | Sexual Molestation |
| I believe I was emotionally abused | CTQ25 | Emotional Abuse Recognition |
| There was someone to take me to medical care if needed | CTQ26 | Medical Care Access |
| I believe I was sexually abused | CTQ27 | Sexual Abuse Recognition |
| My family was a source of strength and support | CTQ28 | Family Support |
| PHQ-9 |  |  |
| Little interest or pleasure in doing things | PHQ1 | Anhedonia |
| Feeling down, depressed or hopeless | PHQ2 | Sad mood |
| Trouble falling or staying asleep, or sleeping too much | PHQ3 | Sleep disturbance |
| Feeling tired or having little energy | PHQ4 | Lack of energy |
| Poor appetite or overeating | PHQ5 | Loss of appetite |
| Feeling bad about yourself | PHQ6 | Guilt |
| Trouble concentrating on things | PHQ7 | Concentration |
| Moving or speaking so slowly | PHQ8 | Motor problems |
| Thoughts that you would be better off dead/of hurting yourself | PHQ9 | Thoughts of death or self-harm |
| GAD-7 |  |  |
| Feeling nervous, anxious or on edge | GAD1 | Nervousness |
| Not being able to stop or control worrying | GAD2 | Uncontrollable worrying |
| Worrying too much about different things | GAD3 | Excessive worry |
| Trouble relaxing | GAD4 | Trouble relaxing |
| Being so restless that it is hard to sit still | GAD5 | Restlessness |
| Becoming easily annoyed or irritable | GAD6 | Irritability |
| Feeling afraid as if something awful might happen | GAD7 | Feeling afraid |

**Table S2 Estimated edge weights of all adolescents**

|  | CTQ1 | CTQ2 | CTQ3 | CTQ4 | CTQ5 | CTQ6 | CTQ7 | CTQ8 | CTQ9 | CTQ11 | CTQ12 | CTQ13 | CTQ14 | CTQ15 | CTQ17 | CTQ18 | CTQ19 | CTQ20 | CTQ21 | CTQ23 | CTQ24 | CTQ25 | CTQ26 | CTQ27 | CTQ28 | PHQ1 | PHQ2 | PHQ3 | PHQ4 | PHQ5 | PHQ6 | PHQ7 | PHQ8 | PHQ9 | GAD1 | GAD2 | GAD3 | GAD4 | GAD5 | GAD6 |
| --- | --- | --- | --- | --- | --- | --- | --- | --- | --- | --- | --- | --- | --- | --- | --- | --- | --- | --- | --- | --- | --- | --- | --- | --- | --- | --- | --- | --- | --- | --- | --- | --- | --- | --- | --- | --- | --- | --- | --- | --- |
| CTQ1 |  |  |  |  |  |  |  |  |  |  |  |  |  |  |  |  |  |  |  |  |  |  |  |  |  |  |  |  |  |  |  |  |  |  |  |  |  |  |  |  |
| CTQ2 | -0.02 |  |  |  |  |  |  |  |  |  |  |  |  |  |  |  |  |  |  |  |  |  |  |  |  |  |  |  |  |  |  |  |  |  |  |  |  |  |  |  |
| CTQ3 | 0.11 | -0.02 |  |  |  |  |  |  |  |  |  |  |  |  |  |  |  |  |  |  |  |  |  |  |  |  |  |  |  |  |  |  |  |  |  |  |  |  |  |  |
| CTQ4 | 0.04 | 0.03 | 0.09 |  |  |  |  |  |  |  |  |  |  |  |  |  |  |  |  |  |  |  |  |  |  |  |  |  |  |  |  |  |  |  |  |  |  |  |  |  |
| CTQ5 | -0.01 | 0.10 | -0.05 | -0.08 |  |  |  |  |  |  |  |  |  |  |  |  |  |  |  |  |  |  |  |  |  |  |  |  |  |  |  |  |  |  |  |  |  |  |  |  |
| CTQ6 | 0.06 | 0.05 | 0.09 | 0.14 | -0.08 |  |  |  |  |  |  |  |  |  |  |  |  |  |  |  |  |  |  |  |  |  |  |  |  |  |  |  |  |  |  |  |  |  |  |  |
| CTQ7 | 0.00 | 0.28 | 0.00 | 0.00 | 0.12 | 0.00 |  |  |  |  |  |  |  |  |  |  |  |  |  |  |  |  |  |  |  |  |  |  |  |  |  |  |  |  |  |  |  |  |  |  |
| CTQ8 | 0.02 | 0.00 | 0.07 | 0.06 | -0.02 | 0.03 | 0.00 |  |  |  |  |  |  |  |  |  |  |  |  |  |  |  |  |  |  |  |  |  |  |  |  |  |  |  |  |  |  |  |  |  |
| CTQ9 | 0.00 | 0.00 | 0.00 | 0.15 | 0.00 | 0.06 | 0.00 | 0.06 |  |  |  |  |  |  |  |  |  |  |  |  |  |  |  |  |  |  |  |  |  |  |  |  |  |  |  |  |  |  |  |  |
| CTQ11 | 0.00 | 0.00 | 0.05 | 0.00 | 0.00 | 0.00 | 0.00 | 0.09 | 0.12 |  |  |  |  |  |  |  |  |  |  |  |  |  |  |  |  |  |  |  |  |  |  |  |  |  |  |  |  |  |  |  |
| CTQ12 | 0.00 | 0.00 | 0.05 | 0.01 | 0.00 | 0.00 | 0.00 | 0.02 | 0.00 | 0.46 |  |  |  |  |  |  |  |  |  |  |  |  |  |  |  |  |  |  |  |  |  |  |  |  |  |  |  |  |  |  |
| CTQ13 | 0.00 | 0.14 | 0.00 | 0.03 | 0.02 | 0.00 | 0.27 | 0.00 | 0.00 | 0.00 | 0.00 |  |  |  |  |  |  |  |  |  |  |  |  |  |  |  |  |  |  |  |  |  |  |  |  |  |  |  |  |  |
| CTQ14 | 0.00 | 0.00 | 0.19 | 0.00 | -0.03 | 0.00 | 0.00 | 0.17 | 0.00 | 0.12 | 0.15 | 0.00 |  |  |  |  |  |  |  |  |  |  |  |  |  |  |  |  |  |  |  |  |  |  |  |  |  |  |  |  |
| CTQ15 | 0.00 | 0.00 | 0.00 | 0.01 | 0.00 | 0.04 | 0.00 | 0.00 | 0.19 | 0.14 | 0.13 | 0.01 | 0.12 |  |  |  |  |  |  |  |  |  |  |  |  |  |  |  |  |  |  |  |  |  |  |  |  |  |  |  |
| CTQ17 | 0.01 | 0.00 | 0.00 | 0.02 | 0.00 | 0.03 | 0.00 | 0.00 | 0.14 | 0.01 | 0.01 | 0.00 | 0.00 | 0.13 |  |  |  |  |  |  |  |  |  |  |  |  |  |  |  |  |  |  |  |  |  |  |  |  |  |  |
| CTQ18 | 0.02 | 0.00 | 0.04 | 0.05 | 0.00 | 0.00 | 0.01 | 0.16 | 0.04 | 0.04 | 0.05 | 0.00 | 0.06 | 0.14 | 0.08 |  |  |  |  |  |  |  |  |  |  |  |  |  |  |  |  |  |  |  |  |  |  |  |  |  |
| CTQ19 | 0.00 | 0.08 | 0.00 | 0.01 | 0.00 | 0.00 | 0.13 | 0.03 | 0.00 | 0.00 | 0.00 | 0.36 | 0.02 | 0.00 | 0.00 | 0.02 |  |  |  |  |  |  |  |  |  |  |  |  |  |  |  |  |  |  |  |  |  |  |  |  |
| CTQ20 | 0.01 | 0.00 | 0.00 | 0.01 | 0.00 | 0.04 | 0.00 | 0.01 | 0.01 | 0.00 | 0.00 | 0.00 | 0.00 | 0.03 | 0.09 | 0.00 | 0.00 |  |  |  |  |  |  |  |  |  |  |  |  |  |  |  |  |  |  |  |  |  |  |  |
| CTQ21 | 0.01 | 0.00 | 0.00 | 0.06 | 0.00 | 0.01 | 0.00 | 0.00 | 0.10 | 0.00 | 0.00 | 0.00 | 0.00 | 0.01 | 0.08 | 0.05 | 0.00 | 0.22 |  |  |  |  |  |  |  |  |  |  |  |  |  |  |  |  |  |  |  |  |  |  |
| CTQ23 | 0.00 | 0.01 | 0.00 | 0.02 | 0.00 | 0.08 | 0.00 | 0.00 | 0.00 | 0.00 | 0.00 | 0.00 | 0.00 | 0.03 | 0.00 | 0.00 | 0.00 | 0.16 | 0.18 |  |  |  |  |  |  |  |  |  |  |  |  |  |  |  |  |  |  |  |  |  |
| CTQ24 | 0.00 | 0.00 | 0.00 | 0.00 | 0.00 | 0.00 | 0.00 | 0.00 | 0.01 | 0.00 | 0.00 | 0.00 | 0.00 | 0.05 | 0.04 | 0.06 | 0.00 | 0.17 | 0.14 | 0.19 |  |  |  |  |  |  |  |  |  |  |  |  |  |  |  |  |  |  |  |  |
| CTQ25 | 0.00 | 0.00 | 0.00 | 0.03 | 0.00 | 0.02 | 0.00 | 0.04 | 0.00 | 0.03 | 0.00 | 0.01 | 0.00 | 0.06 | 0.01 | 0.08 | 0.00 | 0.09 | 0.04 | 0.15 | 0.07 |  |  |  |  |  |  |  |  |  |  |  |  |  |  |  |  |  |  |  |
| CTQ26 | 0.00 | 0.13 | 0.00 | 0.00 | 0.06 | 0.02 | 0.01 | 0.00 | 0.00 | 0.00 | -0.01 | 0.02 | 0.00 | 0.00 | -0.01 | 0.00 | 0.12 | 0.00 | 0.00 | 0.00 | 0.00 | 0.00 |  |  |  |  |  |  |  |  |  |  |  |  |  |  |  |  |  |  |
| CTQ27 | 0.00 | 0.00 | 0.00 | 0.08 | 0.00 | 0.03 | 0.00 | 0.00 | 0.12 | 0.00 | 0.00 | 0.00 | -0.02 | 0.00 | 0.05 | 0.05 | 0.00 | 0.00 | 0.29 | 0.09 | 0.20 | 0.17 | 0.00 |  |  |  |  |  |  |  |  |  |  |  |  |  |  |  |  |  |
| CTQ28 | 0.03 | 0.06 | 0.00 | 0.00 | 0.03 | 0.00 | 0.14 | 0.02 | 0.00 | 0.01 | 0.00 | 0.12 | 0.03 | 0.00 | 0.00 | 0.00 | 0.28 | 0.00 | 0.00 | 0.00 | 0.00 | 0.00 | 0.28 | 0.00 |  |  |  |  |  |  |  |  |  |  |  |  |  |  |  |  |
| PHQ1 | 0.00 | 0.00 | 0.00 | 0.00 | -0.02 | 0.00 | 0.00 | 0.04 | -0.01 | 0.00 | 0.00 | 0.00 | 0.05 | 0.00 | 0.00 | 0.00 | 0.00 | 0.00 | 0.00 | 0.00 | 0.00 | 0.00 | 0.00 | -0.02 | 0.00 |  |  |  |  |  |  |  |  |  |  |  |  |  |  |  |
| PHQ2 | 0.00 | 0.00 | 0.02 | 0.00 | 0.00 | 0.00 | 0.00 | 0.03 | 0.00 | 0.00 | 0.00 | 0.00 | 0.01 | 0.00 | 0.00 | 0.00 | 0.00 | 0.00 | 0.00 | 0.00 | 0.00 | 0.02 | 0.00 | 0.00 | 0.00 | 0.21 |  |  |  |  |  |  |  |  |  |  |  |  |  |  |
| PHQ3 | 0.00 | 0.00 | 0.00 | 0.00 | 0.00 | 0.01 | 0.00 | 0.00 | 0.00 | 0.00 | 0.00 | 0.00 | 0.00 | 0.00 | 0.00 | 0.03 | 0.00 | 0.00 | 0.00 | 0.00 | 0.00 | 0.04 | 0.00 | 0.00 | 0.00 | 0.14 | 0.11 |  |  |  |  |  |  |  |  |  |  |  |  |  |
| PHQ4 | 0.00 | 0.00 | 0.01 | 0.00 | -0.01 | 0.00 | 0.00 | 0.02 | 0.00 | 0.00 | 0.01 | 0.00 | 0.00 | 0.00 | 0.00 | 0.00 | 0.00 | 0.00 | 0.00 | 0.00 | 0.00 | 0.00 | 0.00 | -0.02 | 0.00 | 0.32 | 0.18 | 0.17 |  |  |  |  |  |  |  |  |  |  |  |  |
| PHQ5 | 0.00 | 0.00 | 0.00 | 0.00 | 0.00 | 0.00 | 0.00 | 0.01 | 0.00 | 0.00 | 0.00 | 0.00 | 0.00 | 0.00 | 0.00 | 0.00 | 0.00 | 0.00 | 0.00 | 0.00 | 0.00 | 0.01 | 0.00 | 0.00 | 0.00 | 0.02 | 0.09 | 0.13 | 0.11 |  |  |  |  |  |  |  |  |  |  |  |
| PHQ6 | 0.00 | 0.00 | 0.02 | 0.00 | 0.00 | 0.00 | 0.00 | 0.04 | 0.00 | 0.00 | 0.00 | 0.00 | 0.02 | 0.00 | 0.00 | 0.00 | 0.00 | 0.00 | 0.00 | 0.00 | 0.00 | 0.00 | 0.00 | -0.01 | 0.00 | 0.03 | 0.15 | 0.00 | 0.10 | 0.08 |  |  |  |  |  |  |  |  |  |  |
| PHQ7 | 0.00 | 0.00 | 0.00 | 0.00 | 0.00 | 0.00 | 0.00 | 0.00 | 0.00 | 0.00 | 0.00 | 0.00 | 0.00 | 0.00 | 0.00 | 0.00 | 0.00 | 0.00 | 0.00 | 0.00 | 0.00 | 0.00 | 0.00 | 0.00 | 0.00 | 0.03 | 0.00 | 0.07 | 0.08 | 0.11 | 0.16 |  |  |  |  |  |  |  |  |  |
| PHQ8 | 0.00 | 0.00 | 0.00 | 0.03 | 0.00 | 0.00 | 0.01 | 0.00 | 0.00 | 0.00 | 0.00 | 0.00 | 0.00 | 0.00 | 0.00 | 0.03 | 0.01 | 0.00 | 0.00 | 0.00 | 0.00 | 0.00 | 0.00 | 0.00 | 0.00 | 0.03 | 0.05 | 0.09 | 0.00 | 0.09 | 0.10 | 0.25 |  |  |  |  |  |  |  |  |
| PHQ9 | 0.00 | 0.00 | 0.00 | 0.00 | 0.00 | 0.00 | 0.00 | 0.08 | 0.00 | 0.00 | 0.00 | 0.00 | 0.00 | 0.00 | 0.00 | 0.02 | 0.00 | 0.00 | 0.00 | 0.01 | 0.01 | 0.01 | 0.00 | 0.00 | 0.03 | 0.02 | 0.03 | 0.02 | 0.00 | 0.10 | 0.17 | 0.01 | 0.17 |  |  |  |  |  |  |  |
| GAD1 | 0.00 | 0.00 | 0.00 | 0.00 | 0.00 | 0.00 | 0.00 | 0.01 | 0.00 | 0.00 | 0.00 | 0.00 | 0.04 | 0.00 | 0.00 | 0.00 | 0.00 | 0.00 | -0.02 | 0.00 | 0.00 | 0.00 | -0.01 | 0.00 | 0.00 | 0.01 | 0.11 | 0.00 | 0.07 | 0.00 | 0.01 | 0.00 | 0.00 | 0.00 |  |  |  |  |  |  |
| GAD2 | 0.00 | 0.00 | 0.00 | 0.00 | 0.00 | 0.00 | 0.00 | 0.01 | 0.00 | 0.00 | 0.01 | 0.00 | 0.00 | 0.01 | 0.00 | 0.00 | 0.00 | 0.00 | 0.00 | 0.00 | 0.00 | 0.00 | 0.00 | 0.00 | 0.00 | 0.05 | 0.02 | 0.00 | 0.00 | 0.00 | 0.03 | 0.05 | 0.02 | 0.01 | 0.22 |  |  |  |  |  |
| GAD3 | 0.00 | 0.00 | 0.00 | 0.00 | -0.02 | 0.00 | 0.00 | 0.00 | 0.00 | 0.00 | 0.00 | 0.00 | 0.02 | 0.00 | -0.01 | 0.00 | 0.00 | 0.00 | -0.01 | 0.00 | 0.00 | 0.00 | 0.00 | 0.00 | 0.00 | 0.04 | 0.00 | 0.00 | 0.00 | 0.04 | 0.02 | 0.00 | 0.00 | 0.00 | 0.19 | 0.24 |  |  |  |  |
| GAD4 | 0.00 | 0.00 | 0.00 | 0.00 | 0.00 | 0.00 | 0.00 | 0.00 | 0.00 | 0.01 | 0.00 | 0.00 | 0.00 | 0.00 | 0.00 | 0.00 | 0.01 | 0.00 | 0.00 | 0.00 | 0.00 | 0.00 | 0.00 | 0.00 | 0.02 | 0.00 | 0.03 | 0.03 | 0.00 | 0.01 | 0.04 | 0.02 | 0.00 | 0.07 | 0.05 | 0.14 | 0.17 |  |  |  |
| GAD5 | 0.00 | 0.00 | 0.00 | 0.00 | 0.00 | 0.00 | 0.00 | 0.00 | 0.00 | 0.00 | 0.00 | 0.00 | 0.00 | 0.00 | 0.00 | 0.00 | 0.00 | 0.00 | 0.00 | 0.00 | 0.00 | 0.03 | 0.00 | 0.00 | 0.00 | 0.00 | 0.00 | 0.03 | 0.00 | 0.02 | 0.00 | 0.04 | 0.09 | 0.02 | 0.00 | 0.10 | 0.03 | 0.26 |  |  |
| GAD6 | 0.00 | 0.00 | 0.00 | 0.00 | 0.00 | 0.00 | 0.00 | 0.02 | 0.00 | 0.00 | 0.01 | 0.00 | 0.00 | 0.00 | 0.00 | 0.00 | 0.00 | 0.00 | 0.00 | 0.00 | 0.00 | 0.00 | 0.00 | -0.02 | 0.00 | 0.02 | 0.00 | 0.00 | 0.05 | 0.00 | 0.03 | 0.00 | 0.00 | 0.03 | 0.15 | 0.06 | 0.10 | 0.10 | 0.22 |  |
| GAD7 | 0.00 | 0.00 | 0.00 | 0.00 | 0.00 | 0.00 | 0.00 | 0.00 | 0.00 | 0.00 | 0.00 | 0.00 | 0.00 | 0.00 | 0.00 | 0.00 | 0.00 | 0.00 | 0.00 | 0.00 | 0.00 | 0.00 | 0.00 | 0.00 | 0.00 | 0.01 | 0.01 | 0.00 | 0.00 | 0.00 | 0.07 | 0.02 | 0.02 | 0.00 | 0.02 | 0.07 | 0.21 | 0.06 | 0.22 | 0.19 |

**Table S3 Network-estimator sensitivity analysis**

| Metric | Primary Spearman network | Polychoric sensitivity / between-network comparison |
| --- | --- | --- |
| Non-zero edges | 314 | 547 |
| CTQ8-PHQ9 edge weight | 0.075 | 0.113 |
| CTQ8 two-step bridge expected-influence rank | 9 of 41 | 8 of 41 |
| CTQ8 bridge-strength rank | 7 of 41 | 24 of 41 |
| Edge-presence Jaccard overlap | - | 0.437 |
| Edge-weight correlation between networks | - | Pearson = 0.754; Spearman = 0.628 |
| ridge-rank correlation between networks | - | Two-step expected influence = 0.927; strength = 0.706 |

*Note. Both networks used EBICglasso with γ = 0.5 and n = 2,413. In the polychoric analysis, 486 sparse bivariate cells required continuity correction and the correlation matrix required smoothing.*

**Table S4 Estimator sensitivity of SEM Model 4**

| **Statistic** | **MLR** | **WLSMV** |
| --- | --- | --- |
| CFI / TLI | 0.956 / 0.948 | 0.987 / 0.989 |
| RMSEA / SRMR | 0.063 / 0.027 | 0.046 / 0.027 |
| CTQ8 -> depressive symptoms, Std. beta | 0.439, *p* < 0.001 | 0.385, *p* < 0.001 |
| CTQ8 -> generalized anxiety symptoms, Std. beta | 0.377, *p* < 0.001 | 0.340, *p* < 0.001 |
| Depressive symptoms -> PHQ9, Std. beta | 0.562, *p* < 0.001 | 0.667, *p* < 0.001 |
| Generalized anxiety symptoms -> PHQ9, Std. beta | 0.111, *p* = 0.071 | 0.166, *p* = 0.001 |
| CTQ8 -> PHQ9 direct association, Std. beta | 0.077, *p* = 0.012 | 0.062, *p* = 0.002 |
| Depressive ~~ anxiety residual association | 0.831, *p* < 0.001 | 0.874, *p* < 0.001 |
| Indirect via depressive symptoms, Std. beta | 0.247, *p* < 0.001 | 0.257, *p* < 0.001 |
| Indirect via generalized anxiety symptoms, Std. beta | 0.042, *p* = 0.074 | 0.056, *p* = 0.002 |

*Note. MLR treated item scores as numeric and used robust fit statistics. WLSMV specified all PHQ-9 and GAD-7 items as ordered.*

**Table S5 Measurement-model and discriminant-validity checks**

| **Analysis / index** | **Result** |
| --- | --- |
| Depressive symptom factor CR / AVE | 0.922 / 0.598 |
| Generalized anxiety symptom factor CR / AVE | 0.940 / 0.691 |
| HTMT (Pearson / Spearman sensitivity) | 0.860 / 0.823 |
| Two-factor WLSMV CFA | CFI = 0.954; TLI = 0.946; RMSEA = 0.104; SRMR = 0.022 |
| One-factor WLSMV CFA | CFI = 0.881; TLI = 0.862; RMSEA = 0.167; SRMR = 0.046 |
| Two-factor latent correlation | *r* = 0.900, *p* < 0.001 |

*Note. CR and AVE were calculated from standardized primary Model 4 loadings. HTMT was recalculated from the raw item-level data. One-factor and two-factor measurement models were estimated using WLSMV.*

# Figures

a

**Figure S1 Accuracy and stability of the estimated network.** (a) Accuracy of edge weights estimated via nonparametric bootstrapping. The black dots indicate the bootstrapped mean edge weights, the red line represents the edge weights from the original sample, and the grey area denotes the 95% confidence intervals.

(b) Stability analysis of centrality indices based on case-dropping bootstrap. The blue line shows the expected influence and the red line represents the bridge-expected influence. Shaded areas reflect the corresponding 95% confidence intervals.

**Figure S2 Participant flow.** Of 2,669 questionnaires distributed in selected intact teaching classes, 256 were excluded during pre-analysis screening and 2,413 complete and valid questionnaires were retained. Mutually exclusive reason-specific exclusion counts were not preserved in the archived analytic dataset; no missing core item data remained in the final network or SEM sample.
